# Delineation of the impacts of varying 6-benzylaminopurine concentrations on physiological, biochemical and genetic traits of different olive cultivars under *in vitro* conditions

**DOI:** 10.1093/aobpla/plae038

**Published:** 2024-07-25

**Authors:** Ting Zhao, Sadia Khatoon, Muhammad Matloob Javed, Abdel-Halim Ghazy, Abdullah A Al-Doss, Muhammad Rauf, Taimoor Khalid, Chuanbo Ding, Zahid Hussain Shah

**Affiliations:** College of Traditional Chinese Medicine, Jilin Agriculture Science and Technology College, Jilin, China; College of Traditional Chinese Medicine, Jilin Agriculture Science and Technology College, Jilin, China; Department of Plant Production, College of Food and Agriculture Science, King Saud University, Riyadh 11451, Saudi Arabia; Department of Plant Production, College of Food and Agriculture Science, King Saud University, Riyadh 11451, Saudi Arabia; Department of Plant Production, College of Food and Agriculture Science, King Saud University, Riyadh 11451, Saudi Arabia; Department of Plant Breeding and Genetics, Pir Mehr Ali Shah, Arid Agriculture University, Rawalpindi 46000, Pakistan; Department of Plant Breeding and Genetics, Pir Mehr Ali Shah, Arid Agriculture University, Rawalpindi 46000, Pakistan; College of Traditional Chinese Medicine, Jilin Agriculture Science and Technology College, Jilin, China; Department of Plant Breeding and Genetics, Pir Mehr Ali Shah, Arid Agriculture University, Rawalpindi 46000, Pakistan

**Keywords:** Antioxidant, gene expression, metabolites, PCA, SEM

## Abstract

**Abstract**. The plant growth regulator 6-benzylaminopurine (BAP) is an important component of plant nutrient medium with tendency to accelerate physiological, biochemical and molecular processes in woody plants such as olive. To date, limited knowledge is available on the role of BAP in mediating physiological, biochemical and genetic activities in olives under *in vitro* conditions. To cover this research gap, the current study was conducted with the objective of studying the role of BAP in regulating physiological traits (chlorophyll, CO_2_ assimilation), antioxidant enzymes (superoxide dismutase, catalase and peroxidase), metabolic contents (starch, sucrose and flavonoids) and gene expression (*OeRbcl, OePOD10, OeSOD10, OeCAT7, OeSS4, OeSuSY7, OeF3GT and OeChlH)* under varying concentrations (0, 0.5, 1.5 and 2.5 mg L^−1^) within the provided *in vitro* conditions. The explants obtained from different olive cultivars (‘Leccino’, ‘Gemlik’, ‘Moraiolo’, ‘Arbosana’) were cultured on olive medium (OM) provided with different BAP concentrations using a two-factorial design, and data were analysed statistically. All traits increased significantly under *in vitro* conditions due to increasing concentrations of BAP; however, this increase was more dramatic at 2.5 mg L^−1^ and the least dramatic at 0.5 mg L^−1^. Moreover, correlation, principal component analysis and heatmap cluster analysis confirmed significant changes in the paired association and expression of traits with changing BAP concentration and type of olive cultivars. Likewise, the expression of all genes varied due to changes in BAP concentration in all cultivars, corresponding to variations in physiological and biochemical traits. Moreover, the spectrographs generated via scanning electron microscopy further indicated the variations in the distribution of elements in olive leaf samples due to varying BAP concentrations. Although all cultivars showed a significant response to *in vitro* varying concentrations of BAP, the response of Arbosana was statistically more significant. In conclusion, the current study proved the dynamic impact of the varying BAP concentrations on regulating the physiological, biochemical, and molecular attributes of olive cultivars.

## Teaser

This study investigated the potential role of the different concentrations of 6-benzylaminopurine (BAP) in olive medium on the physiological, metabolic and genetic indicators of olive explants within *in vitro* propagation system. The study proved the dynamic role of BAP in regulating plant growth and development processes in the micropropagation system by affecting various physiological, biochemical and genetic processes. Furthermore, BAP not only consolidates the association of physiological and metabolic processes instead it also regulates the expression of various genes in olive under in vitro conditions. This will further facilitate exploring the new dynamics of olive *in vitro* genetic and molecular studies using different types of plant growth regulators.

## Introduction

Olive (*Olea europaea* L.) is an important plant of the Mediterranean region, mainly used for fruit and oil extraction purposes. Olive propagation using vegetative parts has always remained a potential constraint owing to its high seasonal dependency, long duration and low success rate (Schuch *et al.* 2019). To get rid of this, olive propagation through *in vitro* techniques is considered a better option for sustainable olive production (Dias *et al.* 2022). The process of *in vitro* plant propagation starts with callus induction and ends with shoot induction; therefore, the success of *in vitro* propagation depends on the explant’s response to the supplementation of phytohormones in a nutrient medium (Singh *et al.* 2015). Furthermore, it is really significant to elucidate the different mechanisms both at molecular and physiological levels due to which growth regulators mediate the process of *in vitro* regeneration (Farahani *et al.* 2011; Leva *et al.* 2012). Besides, elucidation of molecular processes regulating somatic development is one of the most important approaches to find the factors that regulate *in vitro* embryogenesis (Bidabadi *et al.* 2020). In the past, the effects of 6-benzylaminopurine (BAP) were widely studied in various micropropagation studies including different plants such as berries, peppermint, summer savoury (*Satureja hortensis* L.) in order to check the effect of BAP on growth traits, biological activities of essential oils and flavonoids accumulation respectively (Shelepova *et al.* 2021; Khlebnikova *et al.* 2022; Turdiyev *et al.* 2023). On the other hand, Bashir *et al.* (2022) have optimized the micropropagation protocol for olive by providing different kinds of ethylene inhibitors in olive medium (OM). Moreover, Regni *et al.* (2023) added neem oil within OM to minimize the use of zeatin in olive somatogenesis.

Olive is preferably cultured on a specially formulated OM with supplementations of growth regulators such as zeatin and BAP (Khatoon *et al.* 2022). In fact, plant hormones act as biostimulants that trigger plant developmental processes via the regulation of various cellular activities and pathways. Cytokinins have the tendency to regulate cell proliferation and division during plant development (Kieber and Schaller 2018). In this perspective, the exogenous supplementation of cytokinins such as BAP and zeatin enhances the process of cell division during the *in vitro* growth process (Toufik *et al.* 2014). Furthermore, cytokinins act as regulators of cytokinin-response genes that are activated in inner or outer cortex cells during the nodule initiation process (Kieber and Schaller 2018). Moreover, under *in vitro* conditions, when plants are grown from vegetative parts, they do not have active metabolic machinery responsible for the growth and differentiation of cells. In this perspective, supplementation of hormones within the *in vitro* propagation system induces rapid growth and differentiation processes leading to early morphogenesis and plant establishment (Peixe *et al.* 2007; Ali *et al.* 2009; Souza *et al.* 2013). Furthermore, BAP boosts the *in vitro* growth of olives by triggering the expression of various growth-related genes (Ramírez-Tejero *et al.* 2020). In plants, physiological and metabolic changes are common indicators of plant growth and development that are modulated by cytokinins. However, till date, few reports are available for explicating the molecular mechanisms regulating physiological and metabolic changes taking place due to the application of cytokinins such as BAP in woody plants (Xuan *et al.* 2020). Somehow, the role of BAP was studied in plants such as carrots in regulating the expression of the genes involved in lignification pathways (Khadr *et al.* 2020). In this perspective, it is important to elucidate the regulatory pattern of various genes triggering physiological and metabolic activities (Shah *et al.* 2017). For instance, magnesium chetalase (*ChlH*) is involved in chlorophyll biosynthesis by stimulating the insertion of Mg in the porphyrin ring; hence, it is important to elucidate the expression pattern of the *OeChlH* gene in olive in association with increased chlorophyll synthesis (Cortleven and Schmülling 2015). Besides, ribulose-1,5-bisphosphate carboxylase-oxygenase (Rubisco) is involved in carbon dioxide fixation; therefore, increased activity of the Rubisco gene (*rbcL*) is an indicator of enhanced physiological activities under the provided conditions (Chen *et al.* 2015). Furthermore, supplementation of biostimulants within a nutrient medium triggers the physiological and metabolic activities within *in vitro* cultured plantlets that can create some sort of oxidative stress (Franzoni *et al.* 2022). However, plant biostimulants enhance the activities of antioxidant enzymes to neutralize the effect of oxidative stress and facilitate the plantlets to retain normal physiological and metabolic activities within the micropropagation system (Yakhin *et al.* 2017). Moreover, the addition of hormones within the culture medium not only helps the plantlets to attain early differentiation but also helps in consolidating the interaction of physiological and biochemical activities through regulation of the expression of various genes (Khatoon *et al.* 2022). For instance, BAP regulates the expression of sucrose synthase (*SuSy*), which triggers the expression of starch synthase (*SS*), to enhance starch accumulation in addition to sucrose production (Kučerová *et al.* 2020). To date, very limited research has been conducted to elucidate the role of BAP in synchronizing the physiological, biochemical and genetic traits of woody plants such as olive. In a previous study, we evaluated the effects of different concentrations of BAP on olive cultivars under *in vitro* conditions at morphological, physiological and biochemical levels. To unravel the prevailing research gap, the current study was conducted with the intention of elucidating the role of BAP in regulating physiological, biochemical and genetic traits in different olive cultivars under varying concentrations under *in vitro* conditions.

## Materials and Methods

The current experiment was conducted using *in vitro* conditions at the plant tissue culture lab of Jillin Agriculture University, China, using four different genotypes of olives such as ‘Leccino’ (Italy), ‘Gemlik’ (Turkey), ‘Moraiolo’ (Italy) and ‘Arbosana’ (Spain). The explants taken from these cultivars were cultured on OM provided with different concentrations (0, 0.5, 1.5 and 2.5 mg L^−1^) of BAP. The effects of varying concentrations of BAP were tested on the physiological, biochemical and genetic traits of olive cultivars using two-factorial arrangements in a completely randomized design, with cultivars as one factor while BAP concentrations as another factor.

### Explant preparation, treatments and propagation

The detached explants from olive cultivars were thoroughly washed for 30 min and divided into nodal segments of 1.5 cm in length. Subsequently, nodal segments were thoroughly sterilized by treating with 70 % ethyl alcohol for 3 min. Furthermore, nodal explants were treated with 15 % sodium hypochlorite for an interval of 15 min and washed three times for 5 min with sterilized distilled water. All sterilization procedures were conducted under aseptic conditions in the laminar airflow chamber. OM (PhytoTech Labs, USA) was prepared based on the formulations of Rugini (1984). Media were supplemented with varying concentrations of BAP (0, 0.5, 1.5 and 2.5 mg L^−1^) (PhytoTech Labs) inside a 300 mL glass bottle, and autoclaving was performed following the method of Chaar-Rkhis *et al.* (2011). Finally, 3–4 explants (nodal segments) were cultured on Rugini OM in glass bottles under a laminar flow chamber. For each treatment, five bottles were used. The *in vitro* cultured explants were placed in a growth chamber under controlled conditions under 2500 lux or 46.25 µmol m^−2^ s^−1^ light intensity, 25 °C temperature and 16 h photoperiod. For biochemical analysis and RNA extraction *in vitro* samples were collected from 60-day-old plantlets.

### Biochemical analysis

Among the biochemical parameters, antioxidant enzymes such as peroxidase (POD), superoxide dismutase (SOD) and catalase (CAT) were measured in terms of their activities following the procedures opted by Shah *et al.* (2022). To attain this objective, 2 g leaf sample of olive plantlets taken from *in vitro* conditions was frozen using liquid nitrogen, and homogenized in 2 mL of ice-cold Tris–HCl buffer having pH7.4 and molarity of 0.1 M. The mixture was adjusted at 4 °C and centrifuged for 15 min at 20 000 *g* for the isolation of the supernatant to measure enzymatic activities. The SOD-kit (Cell Biolabs Inc., USA, Product number 19160-1KT-F) was used to according to manufacturer protocol to assay the activity of SOD, while the POD-kit (Sigma-Aldrich, USA, Product number STA-344) was used to record the activity of POD. Furthermore, a plant CAT-assay kit (Sigma-Aldrich, Product number 219265-1KIT) was used in accordance with the manufacturer’s protocol for assessing the activity of catalases. On the other hand, leaf sucrose was extracted using ethanol and leaf extract was centrifuged at 5000 g for 10 min and quantified for sucrose using high pH anion-exchange chromatography. The sucrose was separated with an anion-exchange column and 12 mM NaOH that was spiked with 1 mM barium acetate serving as an eluent. Furthermore, the acid hydrolysis method was used for the estimation of starch content (Mitchell 1990). The flavonoids were extracted using Zhao *et al.* (2018) procedure and quantified by the colorimetric assay method using rutin as a standard. Besides, chlorophyll content was measured following the procedure used by Arnon (1949), while the net assimilation rate of CO_2_ was measured using an infrared gas analyser-IRGA (ADC BioScientific, UK) following the method opted by Khatoon *et al*. (2022). The data for all biochemical (sucrose, starch, falvonoids, superoxide dismutase, catalase, peroxidase) and physiological parameters (chlorophyll, CO_2_ assimilation) were collected from 60 days old, *in vitro* grown plantlets. The data were collected from five plants of each treatment and averaged for statistical analysis.

### Molecular analysis

For the expression study of genes associated with biochemical and physiological traits such as *OeRbcl, OePOD10, OeSOD10, OeCAT7, OeSS4, OeSuSY7, OeF3GT and OeChlH*, the RNA was extracted in accordance to the procedure opted by Li *et al.* (2019) from randomly selected *in vitro* samples of olive plantlets using Qiagen RNeasy kit (Qiagen, German Town, USA). Furthermore, the cDNA library was constructed following the protocol used by Ahmed *et al.* (2022). For this purpose, 2 μg of RNA was used according to the instructions of the manufacturer. Afterward, qRT-PCR was conducted, and the expression of genes was normalized by using *TaActin1*-expressing gene. During all qRT-PCR analysis, three technical and three biological replicates were used for analysis and confirmation of each expression profile and the relative gene expression for each sample was calculated using a double delta *C*_*t*_ value (Ahmed *et al.* 2022). The primers used in the expression study are mentioned in Table 1.

**Table 1.** Sequences of primers used in relative expression analysis of genes.

Primers	Sequence 5'---3'
*OePOD10*	CGACTACCGCGTGCCTCTT (F) TAGTCCATGGCTAGGCCTA (R)
*OeSOD10*	CGTACCCTGGATTACCGGC (F) GACCGGGTAACGCCCTAG (R)
*OeCAT7*	AGGCCTACCGTACCTAGC (F) GCCCTACGGTACCGGATC (R)
*OeRbcl*	ATG TCA CCA CCA GAG ACT GC (F) GTA AAA TCA AGT CCA CCR CG (R)
*OeChlH*	AGGCTAGGCCTACGACC (F) TCCGGACGGCCTAGCCA (R)
*OeSuSY7*	AGGCCTATGGCCATACA (F) GCCATAGGCCATACCGG (R)
*OeSS4*	GCGGCCTATACGATACG (F) ATCCCGGATCGCGATAC (R)
*OeF3GT*	CCGTACGTAGGTACCCA (F) GCTAGATCCGATGGCAG (R)

### Scanning electron microscopy

For qualitative analysis, the five selected leaf samples of each cultivar from *in vitro* grown olive cultivars at 2.5 mg L^−1^ BAP concentration were kept in EDX system of a scanning electron microscope (SEM) for creating the SEM spectrographs. Moreover, the SEM was equipped with auto-quantification and auto-identification features. Furthermore, in the spectrograph, peak height helped in the quantification of elements and peak position helped in the identification of elements.

### Statistical analysis

The collected data were subjected to statistical analysis using computer-based program statistix8.1 and RStudio version (1.3.959). The Statistix8.1 was used to perform an analysis of variance (ANOVA) at the 5 % probability level and RStudio1.1.456 was used to formulate correlation chart, principal component analysis (PCA) biplot and the heatmap dendrogram. The PCA biplot was constructed using ‘factoextra’ and ‘FactoMineR’ packages of RStudio. Moreover ‘GGally’ and ‘ggplot2’ packages of RStudio were used to construct a Pearson’s correlation chart and ‘pheatmap’; and, ‘complex Heatmap’ packages of RStudio were used to construct Heatmap Dendrogram.

## Results

### Biochemical and physiological traits

All genotypes of olive showed a significant (*P* ≤ 0.05) increase in the activities of antioxidant enzymes (SOD, POD, CAT), amount of starch and sucrose, CO_2_ assimilation (${\text{A}}_{{\text{CO}}_{2}}$), flavonoids and chlorophyll contents due to different concentrations of BAP within OM (Table 2). The activities of the antioxidant enzymes SOD, POD and CAT illustrated a significantly (*P* ≤ 0.05) high increase in all cultivars at a concentration of 2.5 mg L^−1^ followed by 1.5 mg L^−1^, 0.5 mg L^−1^ and control (Table 2). At 2.5 mg L^−1^ concentration, the highest activity of enzymes in terms of enzyme unit was recorded in ‘Arbosana’ (SOD = 36.15, POD = 0.75, CAT = 16.70) followed by ‘Moraiolo’ (SOD = 35.00, POD = 0.71, CAT = 16.00), ‘Gemlik’ (SOD = 34.15, POD = 0.68, CAT = 15.25) and ‘Leccino’ (SOD = 34.15, POD= 0.66, CAT = 15.75). Correspondingly, statistically significant (*P* ≤ 0.05) increase in metabolic contents starch, sucrose and flavonoids in terms of milligram per gram of dry weight (mg g^−1^ DW) was recorded in cultivars ‘Arbosana’ (starch = 0.33, sucrose = 0.37, flavonoids = 1.84) followed by ‘Moraiolo’ (starch = 0.31, sucrose = 0.33, flavonoids = 1.78), ‘Gemlik’ (starch = 0.31, sucrose = 0.35, flavonoids = 1.74) and ‘Leccino’ (starch = 0.30, sucrose = 0.33, flavonoids = 1.71) at BAP concentration 2.5 mg L^−1^. In the same way physiological traits, such as CO_2_ assimilation (${\text{A}}_{{\text{CO}}_{2}}$) and chlorophyll showed a significantly (*P* ≤ 0.05) high increase at BAP concentration 2.5 mg L^−1^ in terms of micromole per metre square per second (μmol m^−2^ s^−1^) and microgram per centimetre square (µg cm^−2^) respectively in genotypes Arbosana’ (${\text{A}}_{{\text{CO}}_{2}}$ = 6.95, chl = 28) followed by ‘Moraiolo’ (${\text{A}}_{{\text{CO}}_{2}}$= 6.85, chl = 27.2), ‘Gemlik’ (${\text{A}}_{{\text{CO}}_{2}}$ = 6.76, chl = 26) and ‘Leccino’ (${\text{A}}_{{\text{CO}}_{2}}$ = 6.73, chl = 26.6). In general among cultivars ‘Arbosana’ followed by ‘Moraiolo’, ‘Gemlik’ and ‘Leccino’ illustrated a maximum increase in biochemical and physiological contents at all concentrations of BAP within *in vitro* propagation system as illustrated in Supporting Information—Fig. S1.

**Table 2.** Effect of different concentrations of BAP on the activity of antioxidant enzymes, biochemical contents and physiological activities of different grown under *in vitro* conditions on olive medium.

BAP (mg L^−1^)	Cultivars	POD (enzyme unit)	SOD (enzyme unit)	CAT (enzyme unit)	Chl (µg cm^−2^)	${\text{A}}_{{\text{CO}}_{2}}$ (μmol m^−2^ s^−1^)	Starch (mg g^−1^ DW)	Sucrose (mg g^−1^ DW)	Flavonoids (mg g^−1^ DW)
0	Leccino	0.23 ± 0.010^mnop^	18.25 ± 0.50^m^	7.50 ± 0.31^hj^	11.0 ± 0.31^o^	3.50 ± 0.10^op^	0.10 ± 0.010^nop^	0.03 ± 0.006^p^	0.81 ± 0.05^p^
	Gemlik	0.21 ± 0.011^o^	18.00 ± 0.55^m^	8.25 ± 0.41^ghi^	12.3 ± 0.19^n^	3.61 ± 0.11^mno^	0.12 ± 0.011^no^	0.05 ± 0.002^o^	0.83 ± 0.06^o^
	Moraiolo	0.24 ± 0.010^mn^	18.45 ± 0.61^m^	7.75 ± 0.35^h^	13.5 ± 0.36^m^	3.72 ± 0.10^mn^	0.11 ± 0.012^n^	0.07 ± 0.001^n^	0.91 ± 0.04^n^
	Arbosana	0.25 ± 0.012^m^	18.75 ± 0.41^m^	8.95 ± 0.41^g^	14.7 ± 0.40^l^	3.85 ± 0.09^m^	0.14 ± 0.013^m^	0.08 ± 0.001^m^	0.95 ± 0.03^m^
0.5	Leccino	0.33 ± 0.011^jkl^	22.55 ± 0.61^l^	10.15 ± 0.43^f^	17.0 ± 0.22^k^	5.40 ± 0.12^kl^	0.19 ± 0.011^l^	0.16 ± 0.001^l^	1.13 ± 0.05^l^
	Gemlik	0.35 ± 0.013^jk^	23.00 ± 0.63^k^	11.25 ± 0.35^e^	18.5 ± 0.40^j^	5.50 ± 0.10^k^	0.21 ± 0.012^ijk^	0.18 ± 0.002^jk^	1.17 ± 0.03^k^
	Moraiolo	0.36 ± 0.015^j^	24.15 ± 0.55^j^	12.25 ± 0.36^d^	19.0 ± 0.50^i^	5.70 ± 0.11^ij^	0.21 ± 0.013^ij^	0.18 ± 0.001^j^	1.24 ± 0.03^j^
	Arbosana	0.32 ± 0.010^i^	25.50 ± 0.60^i^	12.75 ± 0.31^d^	20.0 ± 0.52^h^	5.82 ± 0.12^i^	0.22 ± 0.011^i^	0.22 ± 0.001^i^	1.29 ± 0.05^i^
1.5	Leccino	0.44 ± 0.011^fgh^	28.15 ± 0.62^gh^	14.15 ± 0.33^c^	21.2 ± 0.32^g^	6.31 ± 0.11^fgh^	0.25 ± 0.013^gh^	0.23 ± 0.001^gh^	1.39 ± 0.05^h^
	Gemlik	0.45 ± 0.016^fg^	28.00 ± 0.67^g^	14.00 ± 0.34^c^	22.6 ± 0.53^f^	6.38 ± 0.11^efg^	0.26 ± 0.014^efg^	0.23 ± 0.002^g^	1.43 ± 0.04^g^
	Moraiolo	0.43 ± 0.010^f^	29.00 ± 0.66^ef^	14.25 ± 0.33^c^	23.1 ± 0.91^e^	6.41 ± 0.10^ef^	0.27 ± 0.014^ef^	0.25 ± 0.001^f^	1.47 ± 0.03^f^
	Arbosana	0.51 ± 0.013^e^	29.40 ± 0.71^e^	14.75 ± 0.33^c^	24.2 ± 0.73^d^	6.50 ± 0.09^e^	0.27 ± 0.013^e^	0.31 ± 0.002^e^	1.50 ± 0.04^e^
2.5	Leccino	0.66 ± 0.012^cd^	34.15 ± 0.69^cd^	15.75 ± 0.35^b^	26.6 ± 0.28^c^	6.73 ± 0.11^cd^	0.30 ± 0.012^bcd^	0.33 ± 0.001^bd^	1.71 ± 0.03^d^
	Gemlik	0.68 ± 0.012^c^	34.50 ± 0.43^c^	15.25 ± 0.33^b^	26.0 ± 0.52^c^	6.76 ± 0.09^bc^	0.31 ± 0.013^bc^	0.35 ± 0.002^c^	1.74 ± 0.06^c^
	Moraiolo	0.71 ± 0.010^b^	35.00 ± 0.54^b^	16.00 ± 0.35^a^	27.2 ± 0.95^b^	6.85 ± 0.11^b^	0.31 ± 0.014^b^	0.33 ± 0.001^b^	1.78 ± 0.05^b^
	Arbosana	0.75 ± 0.012^a^	36.15 ± 0.63^a^	16.50 ± 0.33^a^	28.0 ± 0.71^a^	6.95 ± 0.10^a^	0.33 ± 0.014^a^	0.37 ± 0.002^a^	1.84 ± 0.04^a^
	LSD	0.03	1.00	0.75	0.50	0.15	0.021	0.012	0.0081

Parameters illustrating significant difference are indicated in table, while means with difference greater than LSD are significantly different at *P* ≤ 0.05.

### Correlation and PCA

**Figure 1.**
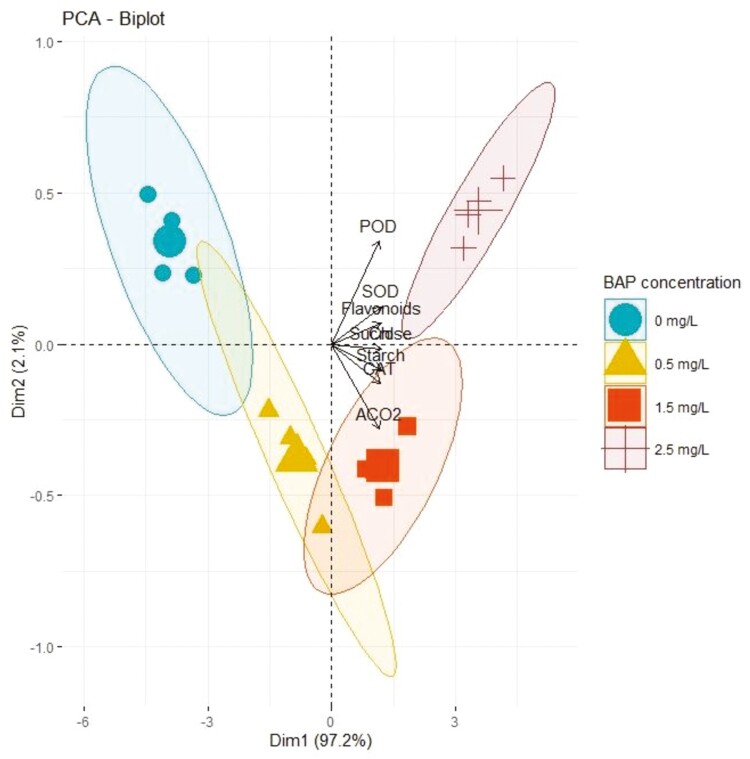
The PCA scattered plot deciphering the magnitude and extent of association of traits as explicated by the length and angle of vectors respectively. Besides, the differential dispersion of eclipses in the plot indicates the differential effect of BAP concentrations (0, 0.5 mg L^−1^, 1.5 mg L^−1^and 2.5 mg L^−1^) on the degree of traits association.

**Figure 2.**
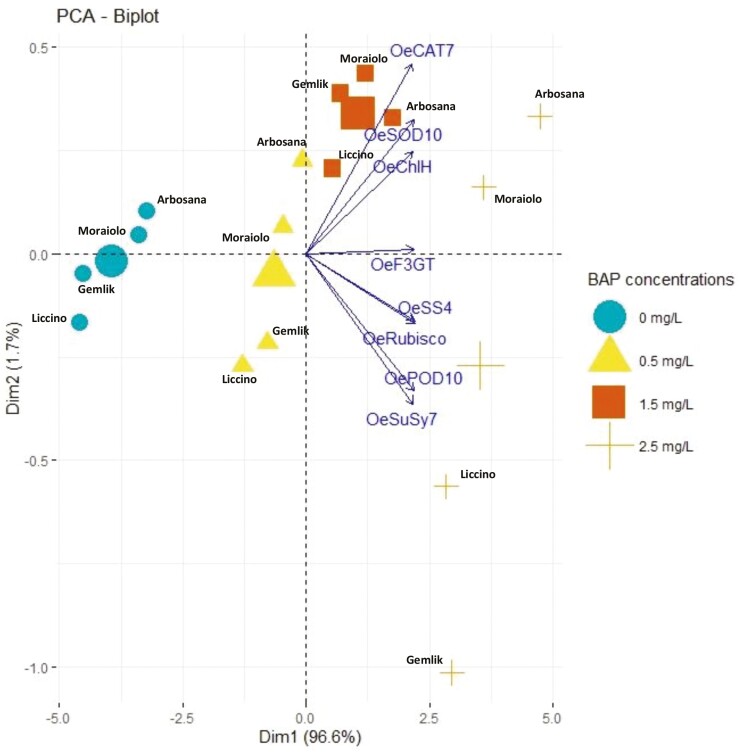
The PCA biplot indicates the differential extent of relative gene expression in olive cultivars under varying concentrations of BAP. The gene lying in the same quadrant of biplot are more closely associated with respect to their expression. Besides, the response of all four olive cultivars varied due to varying concentrations of BAP as revealed by the varying position of concentration with respect to biplot origin.

The correlational graph of all traits represented a significantly high (*P* ≤ 0.001) paired association between all traits in the positive direction as indicated by the blue circles in Supporting Information—Fig. S2. The similar size of circles and colour intensity indicated that traits were associated similarly under different BAP supplementations in OM. As a whole, all traits depicted strong positive paired associations as indicated by high values of correlation coefficient in Table 3. The value of the correlation coefficient among antioxidant enzymes (SOD, POD and CAT), metabolites (starch, sucrose and flavonoids), chlorophyll and CO_2_ assimilation ranged from 0.86 to 1.00 indicated a significantly stronger association of traits under different supplementations of BAP within micropropagation system. Furthermore, PCA revealed the variation of all characters in the same direction as indicated by vector orientation and position with respect to the origin due to different concentrations of BAP in OM (Fig. 1). Besides, difference in length of vectors with respect to origin represented different extents of variation of all traits, while the closeness of vectors represented the strong paired association of traits (Fig. 1). The difference in dispersions of their respective eclipses with respect to origin indicates that all traits varied in different ways due to different concentrations of BAP. This effect was comparatively more significant (*P* ≤ 0.05) at 2.5 mg L^−1^ as illustrated by the high dispersion of eclipse; however, the concentrations of 0.5 and 1.5 mg L^−1^ manifested complementary effect on the variation of all traits as explicated by merged eclipses in PCA biplot (Fig. 1). Besides, in the PCA biplot, all cultivars showed different distributions from origin at all concentrations of BAP. The varying orientation of all cultivars from the biplot vectors indicates that all cultivars showed varying expression of genes even under the same concentrations of BAP (Fig. 2). Moreover, different length of PCA vectors with respect to origin illustrates that all genes are expressed differently in all cultivars. Overall PCA indicates that the expression of genes varied significantly with the type of olive cultivar and amount of BAP concentration.

**Table 3.** The illustration of significance and extent of paired association among antioxidant enzymes (SOD, POD and CAT), metabolites (starch, sucrose and flavonoids), chlorophyll and CO_2_ assimilation.

POD	0.98^***^	0.91^***^	0.95^***^	0.86^***^	0.93^***^	0.94^***^	0.97^***^
	SOD	0.97^***^	0.98^***^	0.94^***^	0.98^***^	0.98^***^	1.00^***^
		CAT	0.99^***^	0.98^***^	0.99^***^	0.98^***^	0.98^***^
			Chl	0.96^***^	0.99^***^	0.99^***^	0.99^***^
				${\text{A}}_{{\text{CO}}_{2}}$ _2_	0.98^***^	0.96^***^	0.95^***^
					Starch	0.99^***^	0.99^***^
						Sucrose	0.99^***^
							Flavonoids

^***^Significant at *P* ≤ 0.001;

^**^Significant at *P* ≤ 0.01;

^*^Significant at *P* ≤ 0.05.

### Genes relative expression analysis

**Figure 3.**
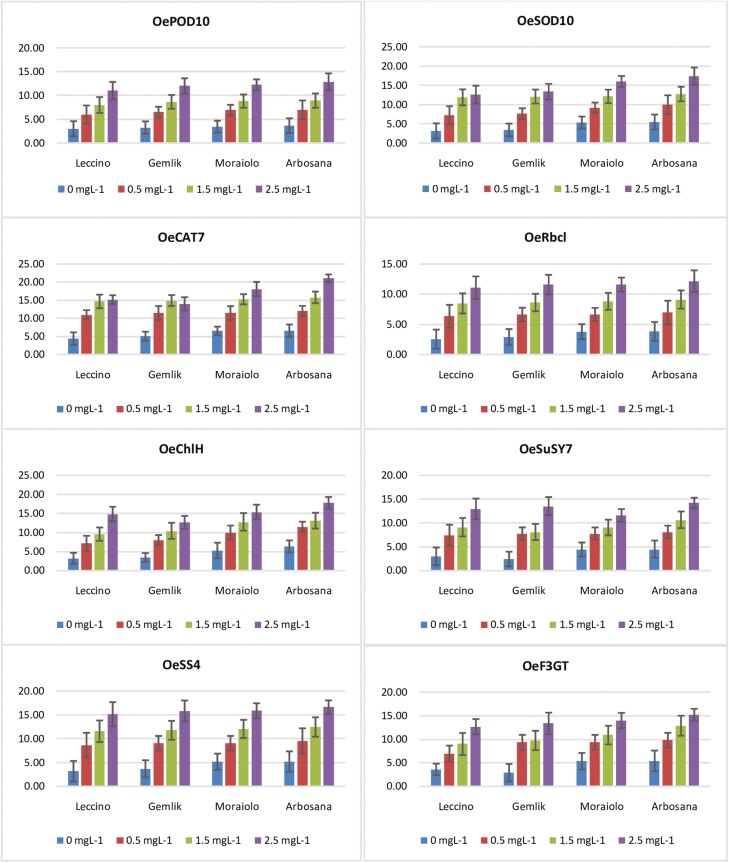
Relative expression of different genes associated with studied traits in different *in vitro* grown olive cultivars within olive medium (OM) supplemented with different concentrations of BAP (0, 0.5 mg L^−1^, 1.5 mg L^−1^and 2.5 mg L^−1^). Bar graphs indicate mean gene expression (*P* ≤ 0.05) and error bars represent the standard deviations when each treatment was tri-replicated.

The expression profiling of *OePOD10* illustrated significantly (*P* ≤ 0.05) different upregulation in all *in vitro* grown olive genotypes under different supplementations of BAP within the OM **[see** Supporting Information**—****Tables S1–S4****]**. The cultivar that had the highest expression of *OePOD10* was ‘Arbosana’, followed by ‘Moraiolo’, ‘Gemlik’, and ‘Leccino’, as shown in Fig. 3.Overall, the relative expression percentage of *OePOD10* was significantly (*P* ≤ 0.05) variable among all *in vitro* propagated olive genotypes under different concentrations of BAP within OM. With maximum increase in expression at 2.5 mg L^−1^ (Arbosana’ = 11.87, ‘Moraiolo’ = 11.25, ‘Gemlik’ = 11.15, ‘Leccino’ = 10.5) and the minimum increase in expression at 0.5 mg L^−1^ (Arbosana’ = 6.95, ‘Moraiolo’ = 6.92, ‘Gemlik’ = 6.87, ‘Leccino’ = 6.63) as compared to no BAP application. The comparative expression analysis of *OeSOD10* revealed statistically significant (*P* ≤ 0.05) variation in all olive genotypes cultured on OM inside the *in vitro* system with different treatments of BAP [**see** Supporting Information**—**Tables S1–S4]. The gene OeSOD10 was significantly upregulated in ‘Arbosana’, followed by ‘Moraiolo’, ‘Gemlik’ and ‘Leccino’ (Fig. 3). Overall, the relative percentage expression of *OeSOD10* was comparatively variable among all olive genotypes due to different supplementations of BAP within OM, with maximum increase in expression at 2.5 mg L^−1^ (Arbosana’ = 17.39, ‘Moraiolo’ = 16.01, ‘Gemlik’ = 13.34, ‘Leccino’ = 12.57) and minimum increase in expression at 0.5 mg L^−1^ (Arbosana’ = 9.93, ‘Moraiolo’ = 9.15, ‘Gemlik’ = 7.62, ‘Leccino’ = 7.18) as compared to zero BAP supplementation (Fig. 3).

The gene *OeCAT7* showed statistically significant (*P* ≤ 0.05) upregulation in all olive genotypes due to varying concentrations of BAP in OM within *in vitro* propagation system [see Supporting Information—Tables S1–S4]. Among cultivars ‘Arbosana’, ‘Moraiolo’, ‘Gemlik’ and ‘Leccino’ showed relatively high percentage expression of *OeCAT7*at all concentrations of BAP (Fig. 3). In general, the expression of *OeCAT7* was comparatively variable among all cultivars at all BAP concentrations, with a maximum increase at 2.5 mg L^−1^ (Arbosana’ = 21.01, ‘Moraiolo’ = 18.04, ‘Gemlik’ = 14.00, ‘Leccino’ = 15.14) and minimum increase at 0.5 mg L^−1^ (Arbosana’ = 12.00, ‘Moraiolo’ = 11.45, ‘Gemlik’ = 11.42, ‘Leccino’ = 10.93) as compared to control condition (Fig. 3).

Relative expression of gene *OeRbcl* showed a significant (*P* ≤ 0.05) difference in all *in vitro* propagated olive genotypes due to different concentrations of BAP within OM [**see** Supporting Information**—**Tables S1–S4]. However, its relative expression was comparatively high in ‘Arbosana’ followed by ‘Moraiolo’, ‘Gemlik’ and ‘Leccino’ as shown in Fig. 3. As a whole, the relative expression percentage of *OeRbcl* was significantly (*P* ≤ 0.05) variable among all *in vitro* propagated olive genotypes under different concentrations of BAP within OM. Besides, the expression of *OeRbcl* recorded maximum increase in all cultivars at 2.5 mg L^−1^ (Arbosana’ = 12.14, ‘Moraiolo’ = 11.58, ‘Gemlik’ = 11.56, ‘Leccino’ = 11.06) BAP level, while minimum increase at 0.5 mg L^−1^ (Arbosana’ = 6.94, ‘Moraiolo’ = 6.62, ‘Gemlik’ = 6.60, ‘Leccino’ = 6.32) BAP application as compared to control.

The relative expression of *OeChlH* increased significantly (*P* ≤ 0.05) with increasing concentrations of BAP within OM under *in vitro* propagation condition [**see** Supporting Information—Tables S1–S4]. Among cultivars ‘Arbosana’ recorded the maximum while ‘Gemlik’ recorded minimum increase in the expression percentage of *OeChlH* at equivalent concentrations of BAP (Fig. 3). Overall, all cultivars illustrated significantly high increase in *OeChlH* expression at 2.5 mg L^−1^ (‘Arbosana’ = 17.77, ‘Moraiolo’ = 15.35, ‘Gemlik’ = 12.84, ‘Leccino’ = 14.81) while significantly low increase at 0.5 mg L^−1^ (‘Arbosana’ = 11.49, ‘Moraiolo’ = 9.96, ‘Gemlik’ = 7.94, ‘Leccino’ = 7.13) BAP concentrationas compared to control treatment.

The gene *OeSuSy7* recorded a significant (*P* ≤ 0.05) increase in expression with increasing concentrations of BAP in OM within *in vitro* propagation system [**see** Supporting Information**—****Tables S1–S4**]. On the other hand among cultivars ‘Arbosana’; followed by ‘Gemlik’, ‘Leccino’ and ‘Moraiolo’ depicted a maximum relative expression percentage of *OeSuSy7* at all BAP concentrations (Fig. 3). Furthermore, expression of *OeSuSy7* (‘Arbosana’ = 14.20, ‘Moraiolo’ = 11.55, ‘Gemlik’ = 13.52, ‘Leccino’ = 12.94) recorded maximum increase at 2.5 mg L^−1^ while minimum increase at 0.5 mg L^−1^ (Arbosana’ = 8.11, ‘Moraiolo’ = 7.74, ‘Gemlik’ = 7.72, ‘Leccino’ = 7.39) as compared to control *in vitro* treatment (Fig. 3).

The relative expression percentage of *OeSS4* demonstrated significant variation (*P* ≤ 0.05) in all olive cultivars due to varying levels of BAP within OM [**see** Supporting Information**—****Tables S1–S4****]**. However, all cultivars did not show any significant difference in the expression of *OeSS4* at corresponding concentrations of BAP (Fig. 3). Besides, all cultivars recorded the maximum increase in the expression of *OeSS4* at 2.5 mg L^−1^ (Arbosana’ = 16.63, ‘Moraiolo’ = 15.87, ‘Gemlik’ = 15.83, ‘Leccino’ = 15.16) while a minimum increase in expression at 0.5 mg L^−1^ (‘Arbosana’ = 9.50, ‘Moraiolo’ = 9.08, ‘Gemlik’ = 9.05, ‘Leccino’ = 8.66) as compared to control (Fig. 3).

Consistent increase in the concentration of BAP in OM under *in vitro* conditions illustrated a significant increase (*P* ≤ 0.05) in the relative expression percentage of *OeF3GT* in all olive genotypes [**see** Supporting Information**—****Tables S1–S4**]. Among cultivars ‘Arbosana’ depicted significantly high while ‘Leccino’ depicted significantly low increase in the expression of *OeF3GT* at corresponding concentrations of BAP (Fig. 3). Besides, BAP concentration 2.5 mg L^−1^ revealed maximum (Arbosana’ = 15.19, ‘Moraiolo’ = 13.98, ‘Gemlik’ = 13.36, ‘Leccino’ = 12.66) while 0.5 mg L^−1^ revealed minimum increase (Arbosana’ = 9.82, ‘Moraiolo’ = 9.37, ‘Gemlik’ = 9.35, ‘Leccino’ = 6.95) in the expression of *OeF3GT* as compared to BAP treatment (Fig. 3).

### Heatmap analysis and traits integration

**Figure 4.**
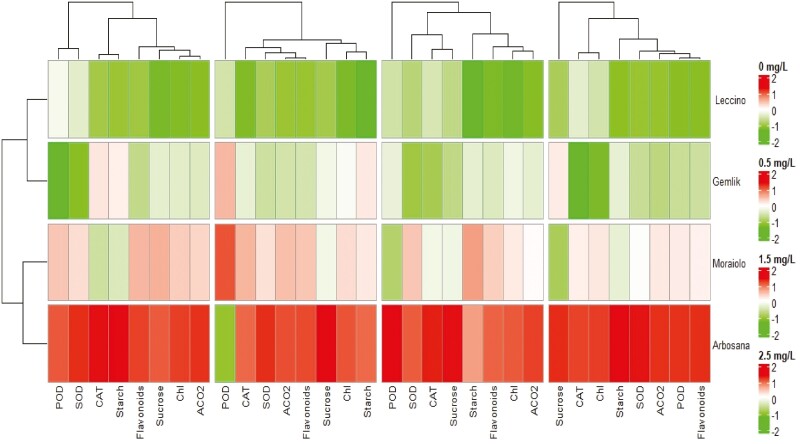
The four clusters (Right to left) of heatmap dendrogram illustrate the differential level of traits expression in different olive cultivars under varying supplementations of BAP (0, 0.5 mg L^−1^, 1.5 mg L^−1^and 2.5 mg L^−1^) in OM, with the highest expression in Arbosana and the lowest expression in Leccino under all levels of BAP supplementations. POD, peroxidase; SOD, superoxide dismutase; CAT, catalase; ${\text{A}}_{{\text{CO}}_{2}}$ CO_2_ assimilation.

**Figure 5.**
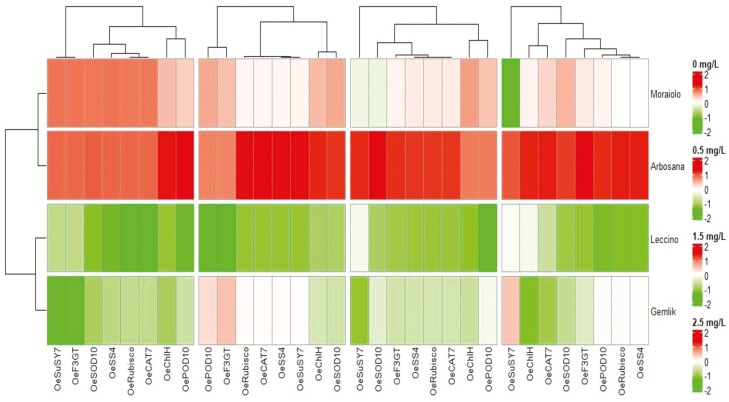
The four clusters (Right to left) of heatmap dendrogram illustrate the differential level of genes expression in different olive cultivars under varying supplementations of BAP (0, 0.5 mg L^−1^, 1.5 mg L^−1^and 2.5 mg L^−1^) in OM, with the highest expression in Arbosana and the lowest expression in Leccino under all levels of BAP supplementations.

**Figure 6.**
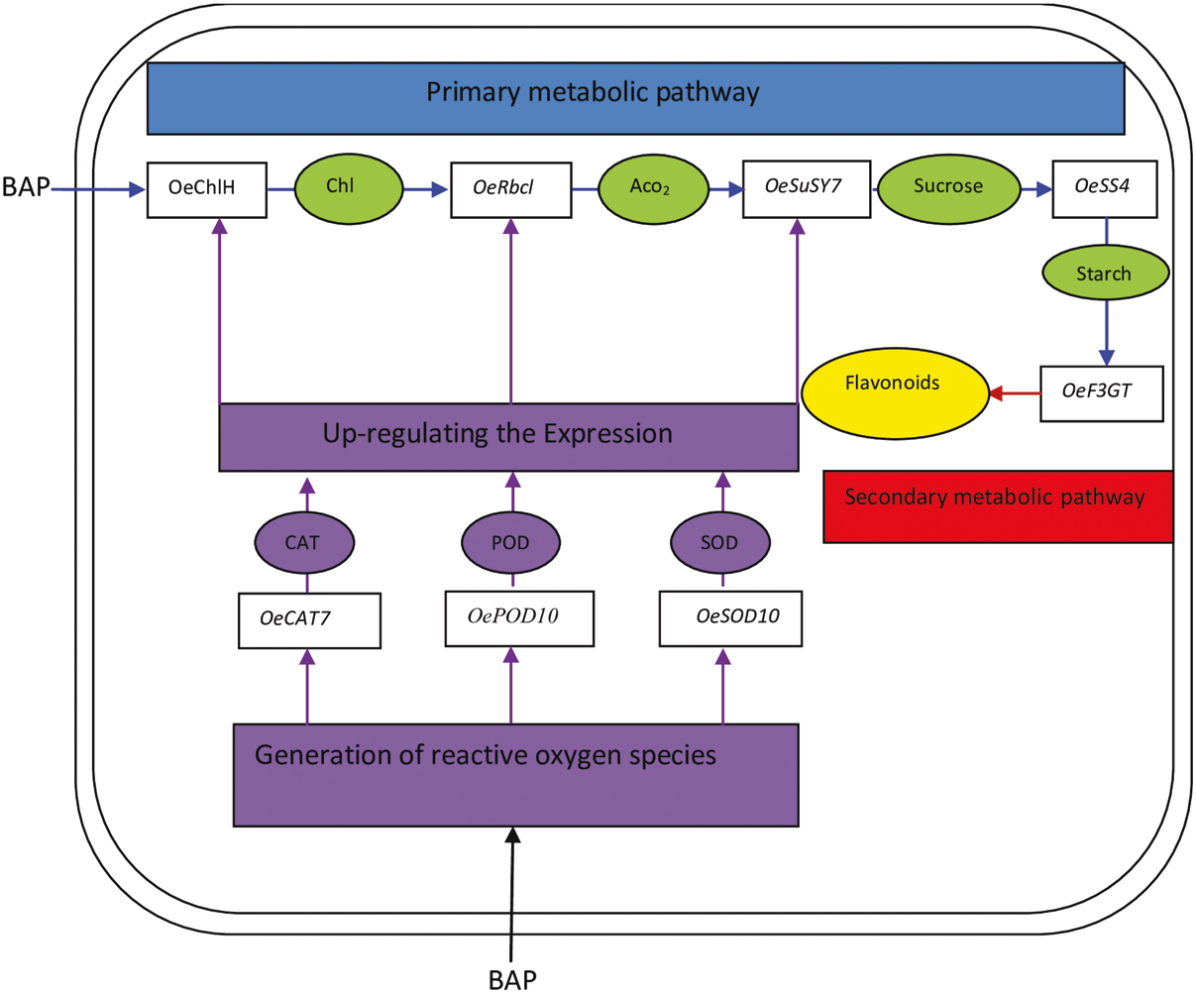
A general model describing how BAP triggers the expression of various genes in olive that determine the expression of physiological and biochemical traits in olive.

Heatmap cluster analysis revealed significant variation in the expression of traits in all olive genotypes due to changing *in vitro* treatments of BAP (Fig. 4). Among all cultivars, ‘Arbosana’ displayed the highest level of trait expression while ‘Leccino’ displayed the lowest level of trait expression (Fig. 4). Correspondingly under all *in vitro* concentrations of BAP, the relative gene expression showed significant variation among all olive cultivars (Fig. 5). Moreover, under all levels of *in vitro* BAP concentrations ‘Arbosana’ showed maximum relative expression of all genes followed by ‘Moraiolo’, ‘Gemlik’ and ‘Leccino’ (Fig. 5). The results from physiological and biochemical analysis were integrated with gene expression in the form of Fig. 6. The results indicate that BAP triggers the expression of genes *OeChlH*, *OeRbcl*, *OeSuSy7* and *OeSS4*, which are involved in a primary metabolic pathway. Increased expression of *OeChlH* enhanced chl synthesis and triggered the expression of *OeRbcl* enhancing the CO_2_ assimilation (${\text{A}}_{{\text{CO}}_{2}}$). Besides, the *OeRbcl* regulates the transcript level of *OeSuSy7* synthesizing the sucrose and regulating the expression of *OeSS4*, which enhances the starch synthesis. The genes of primary metabolic pathway regulate the expression of genes involved in a secondary metabolic pathway, as evident from the expression of *OeF3GT* controlling the production of secondary metabolites, the flavonoids. On the other hand, BAP triggers the the activities of antioxidant enzymes. For instance, the over-expressing genes *OeSOD10, OePOD10* and *OeCAT7*, respectively enhance the activities of SOD, POD and CAT as well as activate the expression of genes involved in primary metabolic pathways.

### SEM micrographs

**Figure 7.**
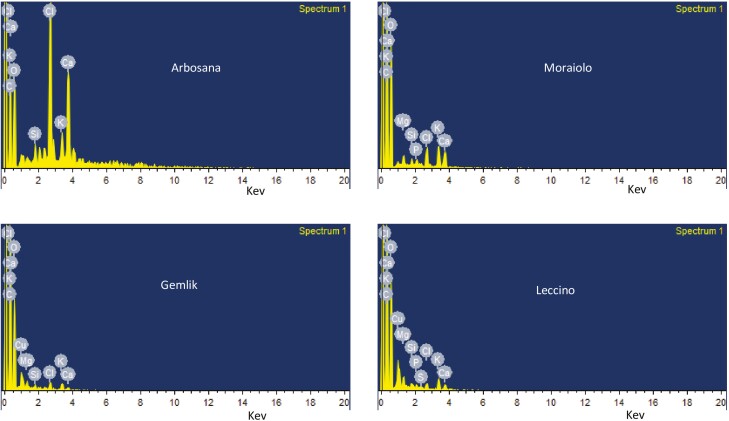
Scanning electron micrographs representing differential elemental distribution in leaf samples of *in vitro* grown olive cultivars at BAP concentration of 2.5 mg L^−1^.

The SEM micrographs obtained at 2.5 mg L^−1^ BAP concentration for the selected leaf samples of all *in vitro* grown olive cultivars depicted clear differences in the assimilation of elements (Fig. 7). The peaks in spectrographs indicated visible differences in the counts of elements that illustrated each cultivar behaves differently under same concentration of BAP. In addition, the broader peak area for ‘Arbosana’ revealed the highest assimilation of nutrients as compared to ‘Moraiolo’, ‘Gemlik’ and ‘Leccino’.

## Discussion

The current study was conducted with the objective to evaluate the effect of different concentrations of BAP on different cultivars of olive propagated in OM using *in vitro* conditions at metabolic, physiological and genetic level. Hormones such as BAP are plant biostimulants having a tendency to trigger physiological and metabolic processes in plants (Yakhin *et al.* 2017). In this perspective, increase in the activities of antioxidant enzymes within *in vitro* grown olive cultivars indicates that plants are responding against some changes imposed to the plant physiological environment due to increased concentrations of BAP (Damanik *et al.* 2019). In fact, antioxidant enzymes regulate various pathways involved in scavenging reactive oxygen species that generates when a plant faces some stress due to excessive growth within nutrient medium (Shah *et al.* 2017). In complementary with these findings, the current study recorded a consistent increase in the activities of antioxidant enzymes such as SOD, POD and CAT with increasing concentrations of BAP within an OM under *in vitro* conditions (Table 2 and Supporting Information**—****Fig. S1**). Furthermore, our findings were consistent with the findings of Vatankhah *et al.* (2010) who noticed an increase in the activities of antioxidant enzymes in *Crocus sativus* owing to increasing levels of BAP. Plant growth regulators tend to trigger plant physiological and metabolic processes by synthesizing photosynthetic apparatus, leading to high chlorophyll levels, increased carbon dioxide (CO_2_) assimilation and metabolite synthesis. (Souza *et al.* 2013). The integrity of physiological and biochemical processes is strengthened by the increased concentrations of BAP, leading to high assimilation of metabolites such as starch and sucrose. (Khatoon *et al.* 2022). Complementary with these findings, the current study recorded a significant increase in the association of biochemical, physiological and metabolic traits under increasing concentrations of BAP [**see** Supporting Information**—****Fig. S2** and Table 3]. On the other hand, paired association traits vary with changing concentrations of BAP, which effects the equilibrium of physiological, biochemical and metabolic processes correspondingly as reviewed by Shah *et al.* (2017). The magnitude and extent of association of all traits (Figs. 1 and 2) may have been affected differently by the different supplementations of BAP in OM, possibly because of this reason. Furthermore, overexpression of genes *OeSOD10, OePOD10* and *OeCAT7* genetically explained the reason for the high activities of SOD, POD and CAT in all *in vitro* grown olive cultivars due to high concentrations of BAP (Fig. 3). Besides, an increase in the level of chlorophyll is highly associated with an increase in CO_2_ fixation resulting in a high accumulation of photosynthates in the form of starch and sucrose (Khatoon *et al.* 2022). It is possible that this is a result of an increase in the activity of enzymes involved in photosynthesis and various metabolic pathways. Furthermore, plant hormones are essential agents that coordinate different physiological and metabolic activities in plants, leading to robust plant growth and development in micropropagation systems (Dia *et al.* 2022). Similarly, Khatoon *et al.* (2022) recorded a dramatic increase in primary and secondary metabolites of all *in vitro* grown olive plantlets due to increasing concentrations of BAP that were maximum at 2.5 mg L^−1^ and minimum at 1.5 mg L^−1^. In fact, BAP supplementation provokes various physiological activities such as high chlorophyll accumulation and increased CO_2_ fixation leading toward the increase in the synthesis of various primary metabolites such as sucrose, glucose and starch and secondary metabolites such as flavonoids, phenols, alkaloids and tannins as reported by Abdelsalam *et al.* (2021) in *Anarrhinum pubescens*. Corresponding to these findings, the current study reported a dynamic increase in chlorophyll content and CO_2_ assimilation in addition to an increase in the synthesis of starch, sucrose and flavonoids (Table 2 and **Supporting Information—****Fig. S1**).

Hormones being plant biostimulants and signalling entities regulate a variety of plant processes by modulating the expression of many genes (Akensous *et al.* 2022). For instance, the highly conserved *Rbcl* gene is involved in the synthesis of Rubisco enzymes regulating photosynthesis via increased CO_2_ fixation as explicated by Chen *et al.* (2015) during their study on *Camellia oleifera.* As per Cortleven and Schmulling (2015), cytokinins like BAP trigger the synthesis of chlorophyll by increasing the expression of the chlorophyll synthesis gene *ChlH*. Likewise, current study reported a significant increase in chlorophyll content and CO_2_ assimilation in all olive cultivars (Table 2 and **Supporting Information—****Fig. S1**) due to an increase in the expression of *OeRbcl* and *OeChlH* with increasing *in vitro* levels of BAP within OM (Fig. 3). Plants are composite entities where all physiological and biochemical processes are strongly correlated and interconnected through various pathways that are regulated by various genes (Shah *et al.* 2017). The biosynthesis of photosynthates such as glucose, sucrose and starch is triggered by BAP’s tendency to upregulate various genes involved in chlorophyll biosynthesis and photosynthesis (Kučerová *et al.* 2020). Overexpression of sucrose synthase (*SuSy*) results in the accumulation of sucrose as an end product of photosynthesis, as reviewed by Stein and Granot (2019). Moreover, high activity of sucrose synthase increases the starch formation by regulating the expression of starch synthase (*SS*) enzyme (Li *et al.* 2013; Cho *et al.* 2020). In parallel with these findings, the current study recorded the increasing relative expression of *OeSuSY7* and *OeSS4* genes, and increasing sucrose and starch content in all olive cultivars due to increasing concentration of BAP in OM (Table 2; **Supporting Information—****Fig. S1** and Fig. 3). Besides, *in vitro* application of BAP increases primary metabolic contents due to enhanced activities of *OeChlH, OeRbcl, OeSuSy* and *OeSS7* that trigger the formation of the substrates involved in secondary metabolic pathway leading towards the synthesis of secondary metabolites such as flavonoids (Shah *et al.* 2017). The increase in expression of candidate genes such as *OeF3GT* is attributed to the high flavonoids contents, as studied by Ramirez-Tejero *et al.* (2020). Overall, the current study proved that *in vitro* addition of BAP to OM alters the biochemical and physiological processes of olive cultivars through regulation of gene expression as indicated in Figs. 4 and 5. Hormones act as a signalling entity and modulate the expressions of genes controlling the plant’s physiological and metabolic processes (Fabregas and Fernie 2021). Therefore, BAP regulates the expression of genes to initiate the primary and secondary metabolic processes that are an integral part of plant growth and development, particularly within *in vitro* conditions (Fig. 6). The phytohormones as a supplement are an integral part of the growth medium. For instance, the cytokinin, such as BAP, defines the role of OM in determining the fate of olive explants (Capelo *et al.* 2010). It has been concluded through various studies the effectiveness of medium along with growth supplements varies from plant to plant and even cultivar to cultivar (Ali *et al.* 2009; Chaari-Rkhis *et al.* 2011; Khatoon *et al.* 2022). Arteta *et al.* (2022) identified the roles of various nutrient elements in achieving a healthy micropropagation system by supplementing different growth regulators, including BAP. In complementary with their findings current study noticed variation in the assimilation of nutrient elements in different types of olive cultivars under the same concentrations of BAP as illustrated in SEM spectrographs (Fig. 7). Additionally, this validates that each cultivar exhibits distinct behaviour under identical micropropagation conditions because of their distinct genetic composition. The results of the previous study (Khatoon *et al.* 2022) were further consolidated by this study, which in-depth integrated BAP effects with genetic determinants of plants. Besides, all four cultivars (Arbosana, Moraiolo, Gemlik and Leccino) used in the present study remained significantly responsive to the applied *in vitro* treatments of BAP as proved through biochemical, physiological and genetic analysis; however, the response of ‘Arbosana’ was statistically more distinct. Furthermore, this study will set a new trend for *in vitro* studies involving optimization of micropropagation protocols through comprehensively evaluating the effects of plant biostimulants at physio-morphic, biochemical and genetic levels.

## Conclusion

Phytohormones are small molecules that have a tendency to regulate every important aspect of plant starting from the development to various responses. In particular, plant hormones impart their specific roles through various signalling pathways regulating the gene expression. In this perspective, present research has shown that plant hormone BAP exerts its impact on plant physiological and metabolic processes through modulating the expression of genes involved in primary and secondary metabolic pathways. In recent research four cultivars of olive showed analogous patterns physiological, biochemical and genetic responses to varying concentrations of BAP; however, the extent of response was different due to variation in the relative expression of genes. In summary, BAP is responsible for regulating plant growth and development processes in the micropropagation system by affecting various physiological, biochemical and genetic processes. Furthermore, BAP not only consolidates the association of physiological and metabolic processes instead it also regulates the expression of various genes in olive under *in vitro* conditions. This will further facilitate in exploring the new dynamics of olive *in vitro* genetic and molecular studies using different types of plant growth regulators.

## Supporting Information

The following additional information is available in the online version of this article –

**Table S1.** Relative expression of various genes in four olive cultivars at 0 mg L^−1^ BAP.

**Table S2.** Relative expression of various genes in four olive cultivars at 0.5 mg L^−1^ BAP.

**Table S3.** Relative expression of various genes in four olive cultivars at 1.5 mg L^−1^ BAP.

**Table S4.** Relative expression of various genes in four olive cultivars at 2.5 mg L^−1^ BAP.

**Figure S1.** Comparative effects of the different concentrations of BAP on the activities of antioxidant enzymes (SOD, POD and CAT), metabolites (sucrose, starch and flavonoids), chlorophyll and CO_2_ assimilation (${\text{A}}_{{\text{CO}}_{2}}$).

**Figure S2.** The correlogram shows different extents of correlation among traits, with positive associations displayed in blue and negative associations displayed in red colour. The legend colour on the right side of correlogram represents correlation coefficients and corresponding colour.

plae038_suppl_Supplementary_Materials

## Data Availability

The data used in the study is available as a part of supporting information.
