# Supplementary material for: Delineation of the impacts of varying 6-benzylaminopurine concentrations on physiological, biochemical and genetic traits of different olive cultivars under in vitro conditions
Source: AoB Plants. 2024 Jul 25;16(4):plae038. doi: 10.1093/aobpla/plae038 (PMC11287765; doi:10.1093/aobpla/plae038)
Supplement: plae038_suppl_Supplementary_Materials [file plae038_suppl_supplementary_materials.docx]

**Table S1. Relative expression of various genes in four olive cultivars at 0 mg/L BAP**

| Genotypes | OeRubisco | OeChlH | OePOD10 | OeSOD10 | OeCAT7 | OeSS4 | OeSuSy7 | OeF3GT |
| --- | --- | --- | --- | --- | --- | --- | --- | --- |
| Leccino | 2.53±1.58 | 3.19±1.45 | 3.10±1.08 | 3.12±1.97 | 4.37±1.73 | 3.16±2.16 | 2.96±1.86 | 3.58±1.24 |
| Gemlik | 2.90±1.31 | 3.38±1.17 | 3.25±1.10 | 3.41±1.63 | 5.01±1.26 | 3.67±1.79 | 2.39±1.49 | 2.89±1.85 |
| Moraiolo | 3.76±1.25 | 5.22±2.07 | 3.45±1.05 | 5.31±1.56 | 6.50±1.16 | 5.15±1.71 | 4.39±1.41 | 5.32±1.77 |
| Arbosana | 3.81±1.56 | 6.31±1.58 | 3.65±0.96 | 5.43±1.95 | 6.59±1.69 | 5.21±2.14 | 4.45±1.82 | 5.39±2.21 |

**Table S2. Relative expression of various genes in four olive cultivars at 0.5 mg/L BAP**

| Genotypes | OeRubisco | OeChlH | OePOD10 | OeSOD10 | OeCAT7 | OeSS4 | OeSuSy7 | OeF3GT |
| --- | --- | --- | --- | --- | --- | --- | --- | --- |
| Leccino | 6.32±1.90 | 7.13±1.98 | 6.63±1.08 | 7.18±2.37 | 10.93±1.28 | 8.66±2.60 | 7.39±2.22 | 6.95±1.69 |
| Gemlik | 6.60±1.12 | 7.94±1.36 | 6.87±1.64 | 7.62±1.40 | 11.42±1.93 | 9.05±1.53 | 7.72±1.31 | 9.35±1.54 |
| Moraiolo | 6.62±1.10 | 9.96±1.82 | 6.92±1.56 | 9.15±1.37 | 11.45±1.90 | 9.06±1.51 | 7.74±1.28 | 9.37±1.23 |
| Arbosana | 6.94±1.96 | 11.49±1.31 | 6.95±1.95 | 9.93±2.45 | 12.00±1.39 | 9.50±2.69 | 8.11±1.29 | 9.82±1.41 |

**Table S3. Relative expression of various genes in four olive cultivars at 1.5 mg/L BAP**

| Genotypes | OeRubisco | OeChlH | OePOD10 | OeSOD10 | OeCAT7 | OeSS4 | OeSuSy7 | OeF3GT |
| --- | --- | --- | --- | --- | --- | --- | --- | --- |
| Leccino | 8.45±1.66 | 9.52±1.75 | 7.33±2.38 | 11.87±2.07 | 14.61±1.81 | 11.57±2.27 | 9.08±1.93 | 8.99±2.35 |
| Gemlik | 8.60±1.45 | 10.41±2.10 | 7.65±1.40 | 12.08±1.81 | 14.87±1.50 | 11.78±1.99 | 8.06±1.69 | 9.75±2.05 |
| Moraiolo | 8.81±1.40 | 12.76±2.32 | 7.81±1.38 | 12.12±1.75 | 15.22±1.42 | 12.05±1.92 | 9.02±1.63 | 10.91±1.96 |
| Arbosana | 9.10±1.50 | 13.07±2.08 | 7.92±2.45 | 12.75±1.87 | 15.74±1.59 | 12.46±2.01 | 10.64±1.75 | 12.88±2.12 |

**Table S4. Relative expression of various genes in four olive cultivars at 2.5 mg/L BAP**

| Genotypes | OeRubisco | OeChlH | OePOD10 | OeSOD10 | OeCAT7 | OeSS4 | OeSuSy7 | OeF3GT |
| --- | --- | --- | --- | --- | --- | --- | --- | --- |
| Leccino | 11.06±1.85 | 14.81±1.89 | 10.51±1.36 | 12.57±2.31 | 15.14±1.20 | 15.16±2.53 | 12.94±2.16 | 12.66±1.62 |
| Gemlik | 11.56±1.62 | 12.64±1.68 | 11.15±1.02 | 13.34±2.02 | 14.00±1.80 | 15.83±2.22 | 13.52±1.87 | 13.36±2.29 |
| Moraiolo | 11.58±1.15 | 15.35±1.90 | 11.25±1.51 | 16.01±1.43 | 18.04±1.98 | 15.87±1.58 | 11.55±1.34 | 13.98±1.63 |
| Arbosana | 12.14±1.77 | 17.77±1.52 | 11.87±1.22 | 17.39±2.21 | 21.01±1.06 | 16.63±1.42 | 14.20±1.07 | 15.19±1.30 |

**Figure S1.** Comparatives effect of the different concentrations of BAP on the activities of antioxidant enzymes (SOD, POD and CAT), metabolites (sucrose, starch and flavonoids), chlorophyll and CO_2_ assimilation (A_CO2_).


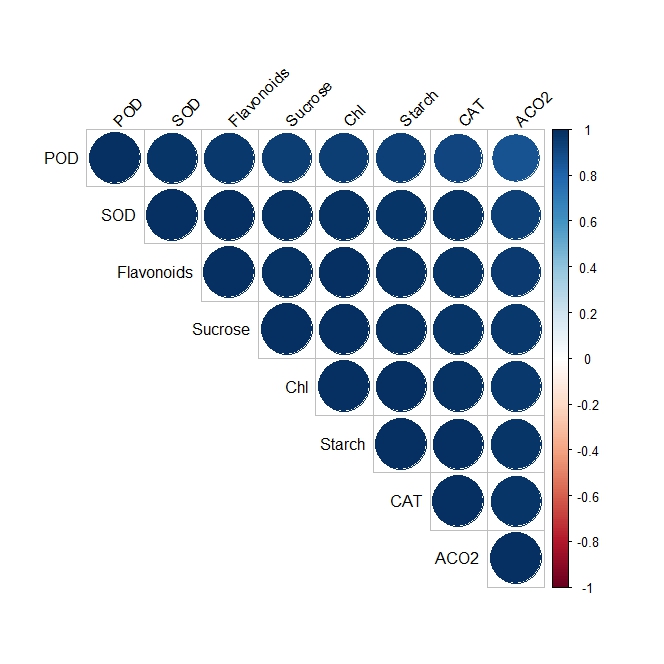


**Figure S2.** The correlogram showing different extent of correlation among traits, with positive association displayed in blue while negative association displayed in red colour. The legend colour on the right side of correlogram represents correlation coefficients and corresponding colour.
